# Evidence for a Constrained Mutational Pathway to High-Level Spectinomycin Resistance in *Neisseria*: RpsE Loop 2 Mutations and Associated Growth Costs

**DOI:** 10.3390/ijms27135971

**Published:** 2026-07-03

**Authors:** Dmitry V. Kravtsov, Dmitry A. Gryadunov, Anastasia A. Anashkina, Boris L. Shaskolskiy

**Affiliations:** Engelhardt Institute of Molecular Biology, Russian Academy of Sciences, 119991 Moscow, Russia; solo13.37@yandex.ru (D.V.K.); grad@biochip.ru (D.A.G.); anastasia.a.anashkina@mail.ru (A.A.A.)

**Keywords:** *Neisseria gonorrhoeae*, *Neisseria* spp., spectinomycin resistance, growth cost, fitness cost, compensatory evolution, *rpsE*, ribosomal protein S5

## Abstract

Antimicrobial resistance in *Neisseria gonorrhoeae* is a global concern. Spectinomycin is unusual in that resistance can emerge rapidly during localized outbreaks yet often disappears from clinical populations after drug withdrawal, suggesting an associated growth cost. To investigate evolutionary routes to spectinomycin resistance, we performed in vitro selection on two *N. gonorrhoeae* strains and two commensal *Neisseria* species. Derived cell lines were characterized by minimum inhibitory concentration (MIC) determination, whole-genome sequencing, growth-kinetics analysis, and molecular modelling of the RpsE (ribosomal protein S5) interface with the ribosome. All high-level resistant isolates (MIC > 2048 mg/L) acquired substitutions or deletions in loop 2 of RpsE. Modelling showed that these mutations perturb the conserved network of stabilizing contacts between RpsE residues Lys25 (Lys23 in *E. coli* numbering) and Lys28 (Lys26), as well as helix 34 nucleotides G922, A923, and C1069 of 16S rRNA, potentially altering the architecture of the spectinomycin-binding site. These mutations were associated with high-level resistance but reduced growth rates, with the resulting growth costs depending on the specific pattern of contact rearrangements. Convergent evolution towards loop 2 mutations supports the existence of a constrained mutational pathway to high-level spectinomycin resistance in the strains and species examined here. This constraint may help explain the rapid decline of resistant variants in the absence of drug pressure and underscores the importance of genomic surveillance.

## 1. Introduction

*Neisseria gonorrhoeae*, the etiological agent of gonorrhea, is estimated to infect approximately 82 million people worldwide each year [1]. The remarkable genetic plasticity of this pathogen underlies its rapid acquisition of antimicrobial resistance [2]. Consequently, strains resistant to previously used therapeutic classes, including penicillins, tetracyclines, fluoroquinolones, and macrolides, as well as to current first-line third-generation cephalosporins, have become widespread. Particularly concerning is the emergence and regional expansion of ceftriaxone-resistant lineages in Southeast Asia over the past five years [3,4,5].

Multiple studies have demonstrated that the acquisition of resistance determinants in *N. gonorrhoeae* is frequently accompanied by measurable fitness costs, as shown for *penA*-35 and Ala501Pro variants [6], the Asian *blaTEM* [7], and the Asp86Asn substitution in *parC* [8]. This raised concerns that the TEM-20 extended-spectrum β-lactamase (ESBL) variants, which are common in *Escherichia coli* [9] and *Salmonella enterica* [10], could eventually emerge in circulating gonococcal populations, given that TEM-20 differs from the prevalent TEM-135 by only a single substitution (Gly238Ser) [11]. However, growth curve analyses have shown that *N. gonorrhoeae* strains expressing TEM-20 exhibit substantially reduced viability compared with TEM-135 carriers, likely explaining why TEM-20 has not been detected in clinical *N. gonorrhoeae* isolates [12].

Cases have also been documented in which resistance determinants impose little or no detectable fitness cost, such as the mosaic *penA-60* allele [13], the ribosomal mutation A2059G [14], or the Ser91Phe and Asp95Asn substitutions in GyrA [8]. In other instances, the associated costs are mitigated by compensatory mutations [15]. Importantly, bacteria tend to acquire compensatory changes rather than revert to the ancestral genotype, which is one reason why reintroducing previously abandoned antibiotics into treatment regimens is often not feasible [16]. One of the few exceptions is spectinomycin, which is currently recommended for gonorrhea treatment in Russia in 2024 [17], in China in 2020 [18], and by the World Health Organization (WHO) in 2024 [1] alongside third-generation cephalosporins.

Spectinomycin is a naturally occurring aminocyclitol antibiotic. Unlike classical aminoglycosides, it lacks nephrotoxicity and ototoxicity and exhibits potent bactericidal activity against *N. gonorrhoeae* [19]. However, it is ineffective against other common sexually transmitted pathogens such as *Chlamydia trachomatis* and *Treponema pallidum* [20]. Resistance to spectinomycin can emerge rapidly but also tends to decline just as quickly once drug pressure is removed, in contrast to resistance to penicillins, tetracyclines, and fluoroquinolones, which remain prevalent despite long-term discontinuation of these agents in gonorrhea treatment [21,22,23,24,25]. Persistence of resistance may be explained not only by low fitness costs but also by the continued use of these drugs for treating co-infections (e.g., chlamydia, syphilis), which maintains selective pressure on *N. gonorrhoeae*. For spectinomycin, however, such co-infection therapy is not a factor, as it is not used against those pathogens. Thus, spectinomycin is one of the few antimicrobials for which reversion to susceptibility at the population level has been documented [21,22,23,26], indirectly suggesting that resistant strains incur substantial biological fitness costs.

Spectinomycin inhibits translation by binding to a specific site on the 16S rRNA within the small (30S) ribosomal subunit. Its classical target is helix 34 (h34), located near the paired nucleotides G1064–C1192 (*E. coli* numbering), which form part of the A-site involved in aminoacyl-tRNA recognition [27,28,29,30]. Binding of spectinomycin disrupts elongation factor G (EF-G)-dependent translocation of peptidyl-tRNA from the A-site to the P-site, thereby arresting elongation [27,28,29,30]. Crystallographic studies of the 70S ribosome have shown that spectinomycin binds within the minor groove of h34 and sterically blocks swiveling of the 30S head, stabilizing a conformation close to the pre-translocation state and preventing normal protein synthesis [31].

Recent structural studies have substantially refined the classical view of how spectinomycin acts on the ribosome. Single-molecule RNA interaction groups analyzed by mutational profiling (RING-MaP) and cryo-electron microscopy (cryo-EM) analyses have demonstrated that its effect is not limited to a local block of translocation. Instead, the antibiotic stabilizes long-range interactions within the 30S subunit that connect the head-swivel axis to the axis of intersubunit rotation, thereby disrupting the coordination between two essential ribosomal motions-head swiveling and intersubunit ratcheting [32]. As noted by Sengupta and colleagues, “Spc binding overstabilizes long-range RNA-RNA contacts that extend 95 Å across the ribosome that connect the pivot for head swiveling with the axis of intersubunit rotation” [32]. These observations are consistent with the concept of “electrostatic levers” and with the broader framework of protein-mediated dynamic networks, in which ribosomal proteins act as coupled regulatory hubs that help coordinate domain motions within the 30S and 50S subunits [33].

Previous studies have shown that resistance in *N. gonorrhoeae* and *N. meningitidis* can arise through mutations in the 16S rRNA (G1064C, C1192T) [34], or through alterations in RpsE, such as deletion of Val27 together with the Lys28Glu substitution, or the Thr24Pro replacement [35,36]. Similar mechanisms have been reported in other bacterial species, including *B. subtilis* [37] and *P. multocida* [38]. Experimental evidence further indicates that RpsE mutations that alter residues within the second loop of ribosomal protein S5 affect not only spectinomycin susceptibility but also translational accuracy and ribosome biogenesis [39], underscoring the functional importance of this structural element.

Despite the long history of spectinomycin use for the treatment of *N. gonorrhoeae*, the determinants of resistance and the associated fitness costs remain incompletely understood, and the mutational pathways leading to resistance are only partially characterized. The rapid disappearance of spectinomycin-resistant strains once the drug is withdrawn suggests that these variants have reduced viability. This hypothesis is consistent with findings from [40], which showed that spectinomycin resistance can be accompanied by a prolonged lag phase in growth curves.

The aim of this study was to model potential pathways of spectinomycin resistance development in vitro and to assess the accompanying changes in growth performance during the emergence of resistant cell lines. The study included a clinical isolate of *N. gonorrhoeae*, the reference strain *N. gonorrhoeae* ATCC 49226, and the reference strains *N. lactamica* ATCC 23970 and *N. sicca* ATCC 9913. The commensal strains were included for two reasons. First, they allowed us to test whether the mutational pathway to high-level spectinomycin resistance is conserved across the genus *Neisseria*. Second, commensal *Neisseria* are recognised reservoirs of antimicrobial resistance determinants and can donate genetic material to *N. gonorrhoeae* through horizontal gene transfer [41].

## 2. Results

### 2.1. Characteristics of Mutant Strains Obtained During Selection

**Figure 1:**
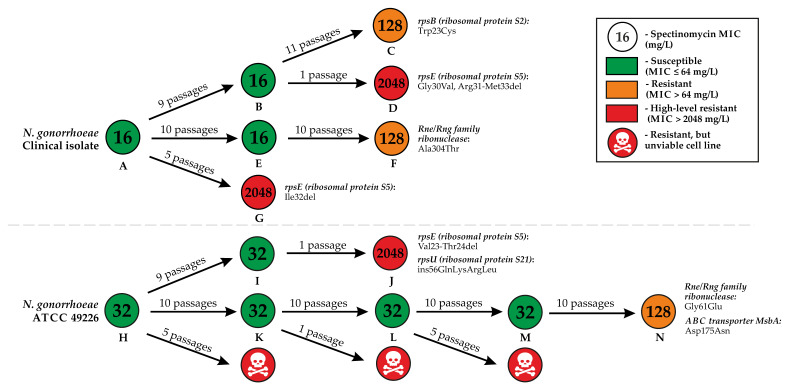
Schematic representation of the derivative cell lines obtained from the *N. gonorrhoeae* clinical isolate and the *N. gonorrhoeae* ATCC 49226 reference strain during stepwise selection on spectinomycin-containing medium. The scheme includes both independent selection series and sequential derivatives obtained during continued passaging.

**Figure 2:**
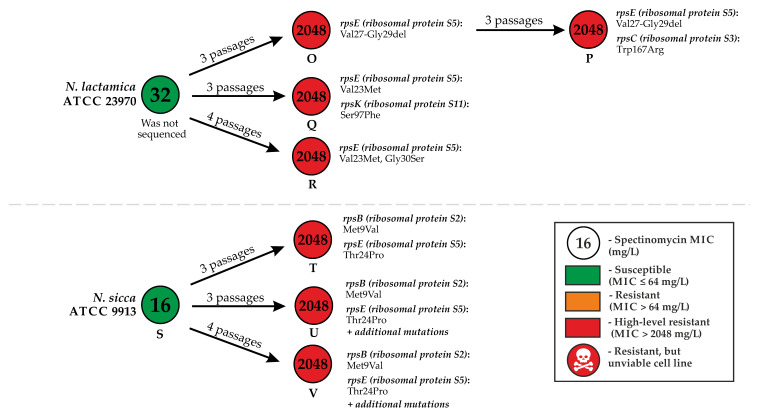
Schematic representation of the derivative cell lines obtained from *N. lactamica* ATCC 23970 and *N. sicca* ATCC 9913 during stepwise selection on spectinomycin-containing medium. The scheme includes both independent selection series and sequential derivatives obtained during continued passaging. For the *N. sicca* derivatives, the available data do not distinguish independent de novo emergence from enrichment of rare pre-existing variants.

A total of 19 derivative cell lines were obtained from the four parental strains through multistep selection on spectinomycin-containing medium. The lineage relationships, together with MIC values and the major genetic changes that emerged, are shown in Figure 1 and Figure 2. The complete set of identified nonsynonymous substitutions and their corresponding MIC values is presented in Table 1.

**Derivative lines of the *N. gonorrhoeae* Clinical isolate.** Six derivative lines were obtained from the parental strain “A” (MIC = 16 mg/L). Line “B” (16 mg/L) did not acquire any detectable nonsynonymous substitutions. Line “C” (128 mg/L) carried the Trp23Cys substitution in ribosomal protein S2 (RpsB), as well as Gly15Asp in a Ppx/GppA phosphatase family protein, an Ala165 frameshift in a site-specific DNA methyltransferase, and Asn61His in pilin. Line “D” (>2048 mg/L) harbored the Gly30Val substitution together with a ΔArg31–Met33 deletion in ribosomal protein S5 (RpsE). Line “E” (16 mg/L) contained only mutations in surface-exposed proteins: a Ser21 frameshift in an opacity family porin and three substitutions in pilin (Lys65Glu, Asp66Asn, Gly69Thr). Line “F” (128 mg/L) carried the Ala304Thr substitution in an Rne/Rng family ribonuclease and Asp259Gly in the endolytic transglycosylase MltG, as well as a Ser21 frameshift in a porin. Line “G” (>2048 mg/L) contained a single-residue deletion, ΔIle32, in RpsE.

**Derivative lines of the *N. gonorrhoeae* ATCC 49226 reference strain.** Seven derivative lines were obtained from the parental strain “H” (MIC = 32 mg/L). Line “I” (32 mg/L) carried the Gly55Asp substitution in a cold-shock protein and an Ala35 frameshift in a sodium/proton antiporter. Line “J” (>2048 mg/L) additionally acquired a ΔVal23–Thr24 deletion in RpsE and an ins56GlnLysArgLeu insertion in ribosomal protein S21 (RpsU). Lines “K”, “L”, and “M” (all 32 mg/L), as well as line “N” (128 mg/L), harbored the Gly61Glu substitution in an Rne/Rng family ribonuclease. Lines “L”, “M”, and “N” shared two mutations: an Ala27 frameshift in an opacity family porin and an intergenic substitution, C1486410T, located between an NCS2 family permease and FtsZ. Line “M” carried additional changes, including a Gln118 frameshift in MtrC, the Thr361Met substitution in the zinc metalloprotease FtsH, and an intergenic insertion (ins1399438AAGC) between Smc and a hypothetical protein. Line “N” (128 mg/L) was also characterized by the Asp175Asn substitution in the lipid A export permease/ATP-binding protein MsbA.

**Derivative lines of the *N. lactamica* ATCC 23970 reference strain.** Four derivative lines with MIC values > 2048 mg/L were obtained from the parental strain (MIC = 32 mg/L). Line “O” carried a triple deletion, ΔVal27–Gly29, in RpsE. Line “P”, in addition to the ΔVal27–Gly29 deletion, acquired the Trp167Arg substitution in ribosomal protein S3 (RpsC) and an intergenic substitution, G1391436A, located between *gdhA* and *pdhR*. Line “Q” contained two substitutions in ribosomal proteins (Val23Met in RpsE and Ser97Phe in RpsK) along with multiple additional substitutions in other genes and intergenic regions. Line “R” carried two substitutions in RpsE (Val23Met and Gly30Ser), the Cys66Tyr substitution in the NADP transhydrogenase PntA, as well as mutations in intergenic regions and genes encoding hypothetical proteins.

**Derivative lines of the *N. sicca* ATCC 9913 reference strain.** Three derivative lines, “T”, “U”, and “V”, with MIC values > 2048 mg/L were obtained from the parental strain “S” (MIC = 16 mg/L). All three lines carried identical mutations: the Thr24Pro substitution in RpsE and the Met9Val substitution in RpsB. These mutations may have arisen de novo in each line or, alternatively, may have been present at low frequency in the ancestral “S” population and subsequently enriched by stepwise exposure to spectinomycin. The present data do not allow these two possibilities to be distinguished.

Line “T” additionally carried the Thr183Asn substitution in the translocation and assembly module subunit TamA. Line “U” was characterized by multiple mutations, including substitutions in metabolic genes (Glu198Asp in NAD(+) synthetase NadE, Ala269Ser in N-acetylmuramoyl-L-alanyl-D-glutamate ligase MurE, and Ala215Ser in the inner membrane protein YccS) along with several frameshift mutations in hypothetical proteins and four intergenic substitutions. Line “V” carried the Ala44Ser substitution in the lipid A export permease/ATP-binding protein MsbA, the Gly147Cys substitution in the ribosome biogenesis GTPase RbgA, as well as numerous intergenic mutations and substitutions in genes encoding hypothetical proteins.

**Figure 3:**
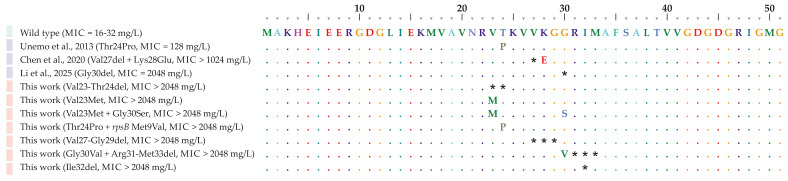
Mutations in loop 2 of RpsE in *Neisseria* cell lines obtained in our study and in those reported by other researchers (Li et al., 2025 [42]) following selection, as well as in clinical isolates (Unemo et al., 2013, Chen et al., 2020 [35,43]) compared with the sequence of the wild-type allele. Dots indicate amino acids identical to the wild type at a given position; letters denote amino acid substitutions; asterisks indicate deletions.

Lines “D”, “G”, and “O” provide the strongest internal evidence for the role of RpsE loop 2 mutations: after stringent filtering of whole-genome sequencing data, each carried a single detectable non-synonymous coding variant, located in RpsE loop 2, and each showed high-level spectinomycin resistance (MIC > 2048 mg/L). Given the particular importance of loop 2 of RpsE, the mutations identified in this region are shown in Figure 3. To provide a comprehensive overview, the figure includes not only the results of our selection experiments but also previously reported polymorphisms in loop 2 of RpsE detected in clinical isolates.

### 2.2. Growth Curves: Relationship Between Growth Rate, Mutations, and Spectinomycin Resistance

**Figure 4:**
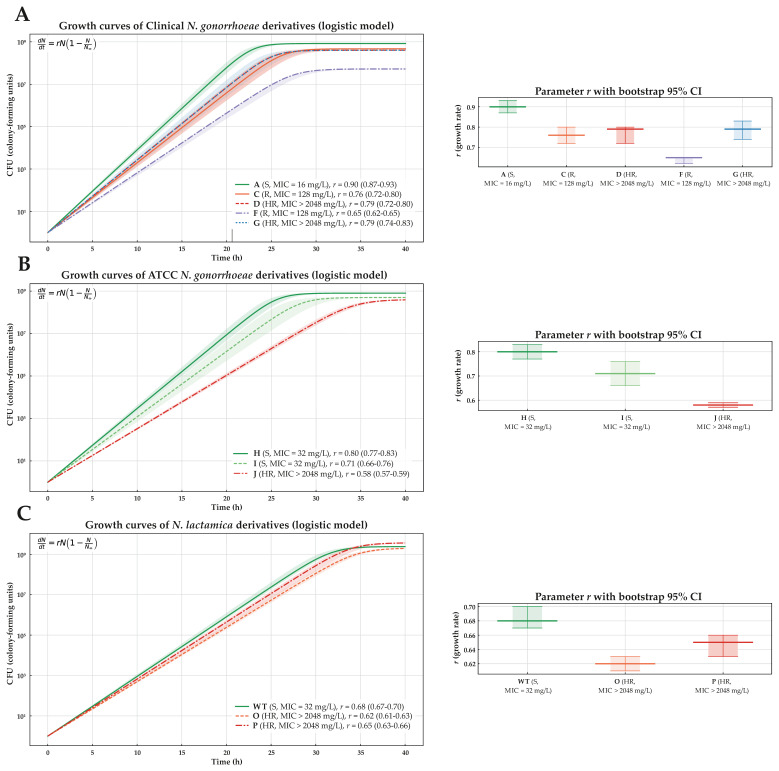
Growth kinetics (left) and parameter estimates of the logistic model (right) for the parental *Neisseria* strains and the derivative cell lines obtained through selection on spectinomycin-containing medium. Solid lines represent point estimates corresponding to the median values of the growth rate parameter *r*, and shaded confidence bands indicate the 95% confidence intervals reflecting variation in *r* between *r*_min_ and *r*_max_ at a fixed *N_∞_*. The panels on the right show boxplots visualizing the distribution of *r* for each cell line: the central line represents the median, and the whiskers correspond to the 95% confidence interval. Labels beneath the boxplots list the key mutations associated with changes in resistance level. (**A**) Lines of the *N. gonorrhoeae* Clinical isolate. (**B**) Lines of the *N. gonorrhoeae* ATCC 49226 reference strain. (**C**) Lines of the *N. lactamica* ATCC 23970 reference strain.

To understand how the identified mutations affect cellular physiology, we examined the growth kinetics of the derived cell lines. An inverse parameter-estimation approach was applied to the experimental growth data to quantify growth-rate reductions and obtain estimates of the logistic-model parameters. These estimates were then used to reconstruct the kinetic growth curves of the *Neisseria* cell lines (Figure 4) and to compute confidence intervals for the inferred parameters. Comparison of the growth rate parameter *r* with the genotypes and resistance levels indicated a relationship between mutation patterns, MIC values, and the growth cost.

The parental *N. gonorrhoeae* lines Clinical “A” and ATCC 49226 “H” exhibited high growth rate values (0.87–0.93 and 0.77–0.83, respectively), consistent with normal physiology and the absence of constraints on growth. These values served as the baseline for assessing the impact of spectinomycin resistance on growth performance.

At intermediate stages of selection, when the MIC increased only to 128 mg/L and the mutations affected genes not directly related to the spectinomycin target (for example, *rpsB*, *rne*, *mltG*, porin genes, and pilin genes), a moderate reduction in the growth rate parameter *r* was observed, down to 0.62–0.76. Such lines (“C” and “F”) represent adaptive states that provide partial increases in resistance through auxiliary pathways that do not disrupt the structure of helix 34 of the 16S rRNA.

A transition to high-level resistance (MIC > 2048 mg/L) occurred only when amino acid substitutions or deletions emerged in loop 2 of ribosomal protein S5 (RpsE), spanning residues 19–33. Across the strains examined, high-level resistance was consistently associated with mutations in RpsE loop 2, including Val23Met, Val23Met + Gly30Ser, ΔVal23–Thr24, Thr24Pro, ΔVal27–Gly29, ΔIle32 and Gly30Val + ΔArg31–Met33. These mutations consistently resulted in a marked reduction in the growth rate parameter *r* to 0.58–0.79. The largest reduction in growth rate was observed in the ATCC line “J” (ΔVal23–Thr24), where *r* declined from 0.8 to 0.58. In contrast, the highly resistant Clinical lines “D” (Gly30Val + ΔArg31–Met33) and “G” (ΔIle32) showed a milder decrease in *r* (from 0.9 to 0.79), reflecting differences in the size and position of the deletions. A similar pattern was observed in *N. lactamica* (“O”, “P”): acquisition of high-level resistance (MIC > 2048 mg/L) was accompanied by a reduction in *r* from 0.68 to 0.62–0.65. Line “P” (*r* = 0.65), which differs from its ancestral line “O” (*r* = 0.62) by the Trp167Arg substitution in RpsC, exhibited a slight recovery of growth, indicating a compensatory effect of this mutation.

### 2.3. Cross-Resistance to Gentamicin and Kanamycin

To assess whether the selected mutations affected susceptibility to aminoglycosides, MIC values for kanamycin and gentamicin were determined for all derived cell lines (Table 2). The parental *N. gonorrhoeae* strains Clinical “A” and ATCC “H” were susceptible to kanamycin (4 and 16 mg/L, respectively) and gentamicin (4 and 4–8 mg/L). Acquisition of spectinomycin resistance was not accompanied by substantial changes in susceptibility to either drug. The largest increase in MIC was observed in line ATCC “J”, where the kanamycin MIC rose to 32–64 mg/L and the gentamicin MIC to 16 mg/L. However, these values remain below the clinical resistance thresholds (>64 mg/L for kanamycin and >16 mg/L for gentamicin). Moderate increases in kanamycin MIC (up to 16–32 mg/L) were also noted in lines “D”, “I”, “L”, “M”, “P” and all *N. sicca* derivatives. An increase in gentamicin MIC to 16 mg/L was recorded for lines “P”, “U”, and “V”. Under the provisional gentamicin and kanamycin thresholds proposed in the literature [44,45], none of the spectinomycin-resistant derivatives showed MIC increases that would be classified as clinically relevant cross-resistance to these aminoglycosides.

### 2.4. Molecular Modeling of Mutations in RpsE Loop 2

To evaluate the structural consequences of the amino acid substitutions and deletions in loop 2 of ribosomal protein S5 identified in the lines with high-level spectinomycin resistance (MIC > 2048 mg/L), homology modeling of the corresponding RpsE variants was performed using the MOE 2019.0102 software package. The crystal structure of the *E. coli* 30S ribosomal subunit in complex with spectinomycin (PDB ID: 4V56) was used as the template, as it provides a high-resolution representation of the S5–h34 interface and the antibiotic-binding site. Due to the use of this *E. coli* template for all structural analyses, 16S rRNA nucleotide positions are reported in *E. coli* numbering throughout; amino acid positions in RpsE are given in *N. gonorrhoeae* numbering with the *E. coli* equivalent in parentheses (as introduced in the Abstract and used in Section 3.1 and Section 3.2).

**Figure 5:**
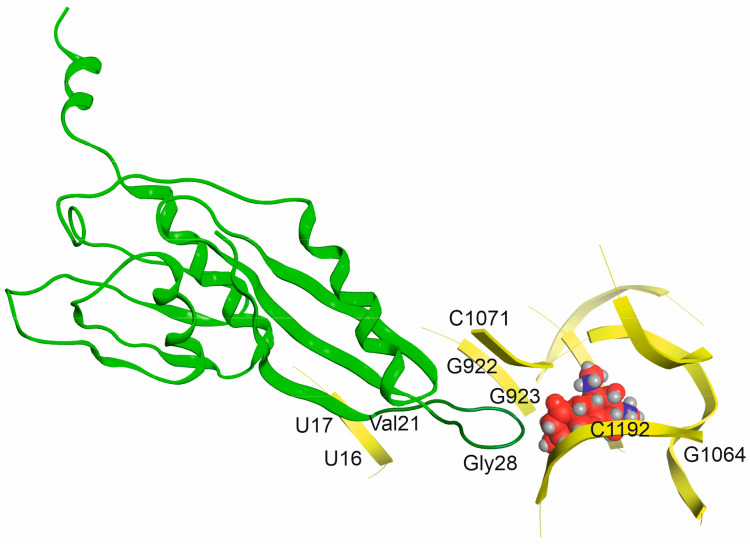
Structure of protein S5 (shown in green, with loop 2 in dark green), fragments of the 16S rRNA (shown in yellow), and spectinomycin (colored red, blue, and gray for carbon, nitrogen, and hydrogen atoms, respectively). Amino acid and nucleotide positions are given according to *E. coli* numbering.

A fragment of the 70S ribosome structure containing protein S5, the h34 segment of the 16S rRNA, and the spectinomycin molecule is shown in Figure 5. The figure highlights the amino acid residues of loop 2 for which the models revealed changes in interaction energies, as well as the 16S rRNA nucleotides that form the contact surface with S5. Among these are the classical rRNA determinants of spectinomycin resistance, G1064 and C1192 (*E. coli* numbering), mutations in which (G1064C, C1192T) have previously been associated with high-level resistance.

For the wild-type (WT) protein and the seven mutant variants corresponding to lines “D”, “G”, “J”, “O”, “Q”, “R”, and “T” we calculated two integral descriptors. These included the polar/hydrophobic component of the solvation free energy (GBVI) and the total contact energy (TCE). TCE was defined as the sum of all pairwise interaction energies between S5 and the surrounding components of the 30S subunit (16S rRNA, neighboring proteins, water molecules, and spectinomycin). Negative values of both parameters indicate a stable interface; the more negative the TCE, the stronger the overall stabilization of the protein–environment interface. The results are summarized in Table 3.

**Figure 6:**
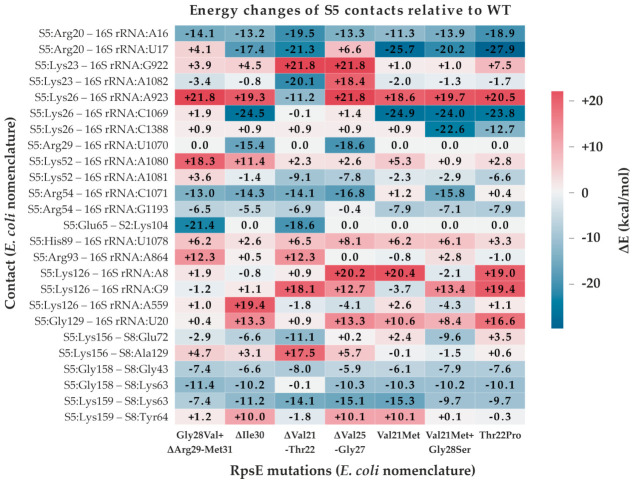
Heatmap of interaction-energy changes (ΔE, kcal/mol) for the 25 S5 contacts with the largest mean absolute deviation from the WT. Cell values represent the “mutant–WT” difference: negative values (blue) indicate increased stabilization, whereas positive values (red) indicate destabilization. Contact labels are given in the format “S5:Residue–Partner.” Amino-acid and nucleotide positions follow *E. coli* numbering.

The GBVI and TCE values for all models remained within a narrow range: GBVI varied from −49.4 to −53.5 kcal/mol, and TCE from −743.7 to −873.9 kcal/mol. None of the mutants showed a dramatic deviation from the WT in these integral descriptors, which is consistent with the local nature of the mutations and with the limited accuracy of molecular mechanics energy calculations. To identify specific structural rearrangements underlying these effects, changes in the energies of individual pairwise contacts were analyzed. All contacts were ranked by the absolute magnitude of their energy change relative to WT (ΔEnergy = Energy_mutant − Energy_WT). The twenty-five contacts with the largest mean absolute change (MeanAbsChange) are shown in the heatmap (Figure 6). The complete list of S5-ribosome contacts with their interaction energies is provided in the Supplementary Materials (Supplementary Table S2).

Analysis of the interaction energy profiles suggested three major groups of contacts that undergo systematic rearrangements in response to mutations in RpsE loop 2 (numbering according to *E. coli*).

**Contacts with the 16S rRNA region adjacent to helix h34.** Although nucleotides U921, G922, and A923 are not part of h34 itself (helix 34 encompasses nucleotides 1046–1067 and 1189–1211), they lie in close proximity to the spectinomycin-binding site and form stabilizing interactions with Lys23 and Lys26 of S5 in the WT structure. In all mutant variants, these interactions undergo pronounced rearrangements. The S5:Lys23–16S rRNA:G922 contact, which is strongly stabilizing in the WT (−21.8 kcal/mol), loses its stabilizing role in ΔVal25–Gly27 and ΔVal21–Thr22, decreasing to values near zero and redirecting Lys23 toward G15 (−24.9 kcal/mol) and A1082 (−38.4 kcal/mol), respectively (Supplementary Table S2).

The most pronounced interaction-energy changes occur in the S5:Lys26–16S rRNA:A923 pair. In the WT, this contact is strongly stabilizing (−21.8 kcal/mol) (Supplementary Table S2), but in all mutants except ΔVal21–Thr22 it becomes markedly weakened or is lost entirely (ΔE = +18.6 … +21.8 kcal/mol) (Figure 6). In the Val21Met, Val21Met + Gly28Ser, ΔIle30, and Thr22Pro variants, stabilization of the alternative S5:Lys26–16S rRNA:C1069 contact increases substantially (ΔE = −23.8 … −24.9 kcal/mol), indicating a redirection of Lys26 toward a different nucleotide. In contrast, the Gly28Val + ΔArg29–Met31 mutant redirects Lys26 to U1070 (−21.2 kcal/mol), a contact absent in both the WT and all other mutants. Notably, the direct interaction between S5 and spectinomycin (S5:Lys26–SCM:1661), which is nearly neutral in the WT, becomes strongly stabilizing in this same mutant (−13.0 kcal/mol) (Supplementary Table S2), directly indicating an altered mode of antibiotic binding. Collectively, these rearrangements are predicted to disrupt the ability of S5 loop 2 to maintain the h34 conformation required for efficient spectinomycin binding to the 16S rRNA.

**Stabilization of the S5-rRNA interface.** In addition to local effects, the mutations trigger a cascade of changes in more distant regions of the 16S rRNA. For example, the S5:Arg54–16S rRNA:C1071 interaction, which is essentially absent in the WT (ΔE ≈ 0 kcal/mol), becomes noticeably stabilizing in all mutants except Val21Met and Thr22Pro, reaching −16.8 kcal/mol in ΔVal25–Gly27. The S5:Lys52–16S rRNA:A1080 contact, which is stable in the WT, is markedly weakened in ΔIle30 and Gly28Val + ΔArg29–Met31 (ΔE = +11.4 and +18.3 kcal/mol, respectively).

Moderate stabilization of the S5:Arg20–16S rRNA:A16 interaction in the WT (−9.8 kcal/mol) becomes substantially stronger in all mutants (ΔE = −11.3 … −19.5 kcal/mol). The interaction of Arg20 with the neighboring nucleotide U17 is also strongly enhanced in all variants (ΔE = −17.4 … −27.9 kcal/mol), except for ΔVal25–Gly27 and Gly28Val + ΔArg29–Met31. These findings indicate that local perturbations within loop 2 propagate through the S5–rRNA contact network over considerable distances.

**Protein–protein contacts between S5 and S8/S2.** Disruption of native rRNA interactions is also reflected in altered contacts with neighboring proteins. The S5:Gly158–S8:Lys63 and S5:Lys159–S8:Lys63 pairs, which are weakly expressed in the WT, become markedly stabilizing in all mutants except ΔVal21–Thr22 (reaching −11.4 and −15.1 kcal/mol, respectively). This indicates a shift in S5 toward S8 and the formation of new protein–protein contacts that may partially compensate for the loss of native geometry. In addition, S5:Glu64 acquires strong stabilization with S2:Lys104 in the ΔVal21–Thr22 and Gly28Val + ΔArg29–Met31 mutants (−18.6 and −21.4 kcal/mol), suggesting that protein S2 becomes involved in the adaptive response to structural perturbations.

## 3. Discussion

### 3.1. Mutations Associated with Spectinomycin Resistance

High-level spectinomycin resistance in all examined strains arose predominantly through mutations located in loop 2 of ribosomal protein S5 (RpsE), including Val23Met, Val23Met + Gly30Ser, Thr24Pro, ΔVal23–Thr24, ΔVal27–Gly29, ΔIle32, and Gly30Val + ΔArg31–Met33. This convergence of evolutionary trajectories is consistent with current structural and functional models of spectinomycin action, which propose that the antibiotic binds within the minor groove of helix 34 (h34) of the 16S rRNA at the “neck” of the 30S subunit and stabilizes a partially rotated head conformation, thereby interfering with the normal tRNA translocation cycle [31]. Our analysis of S5 interaction energies showed that all of these mutations are predicted to disrupt the balanced network of contacts between loop 2 and the nucleotides of h34. In the WT structure, Lys25 (Lys23 in *E. coli* numbering) and Lys28 (Lys26) form stabilizing interactions with G922 (−21.8 kcal/mol), A923 (−21.8 kcal/mol), and C1069 (−1.4 kcal/mol), thereby fixing loop 2 in a conformation that supports the architecture of the antibiotic-binding site. In the mutants, this network collapses. For example, the Lys25(Lys23)–G922 interaction becomes destabilizing in the ΔVal23–Thr24 (ΔVal21–Thr22) variant (+21.8 kcal/mol), whereas in the Val23Met (Val21Met) variant the position of Lys28 (Lys26) shifts from A923 to C1069 (−26.3 kcal/mol). Such rearrangements likely impair the ability of S5 to maintain the h34 conformation required for high-affinity spectinomycin binding, which may help explain the sharp increase in MIC. These structural models should be interpreted as a mechanistic hypothesis rather than direct proof of the conformational states adopted by the mutants in vivo. This limitation is particularly relevant for deletion variants in loop 2, whose backbone conformations are difficult to predict confidently by homology modelling alone. For this reason, we present a targeted structural comparison for the representative point mutation Val23Met (Val21Met in *E. coli* numbering; Supplementary Figure S1), whereas the broader structural analysis is based on comparative contact-energy changes across all mutants (Figure 6 and Supplementary Table S2).

A similar mutational spectrum was recently reported in *E. coli*: all spectinomycin-resistant clones obtained through selection on spectinomycin-containing medium carried mutations within a narrow region of RpsE homologous to loop 2 of protein S5 [46]. Mutations in this locus have also been described in *Bacillus subtilis* [37] and *Pasteurella multocida* [38], indicating that this mechanism is conserved across diverse bacterial species.

Loop 2 of RpsE lies in direct proximity to h34 and participates in interactions that are critical for shaping the local spectinomycin-binding interface, as supported by both structural and genetic evidence [38,47]. In addition, h34 forms part of a dynamic node that coordinates head swiveling and intersubunit ratcheting and is highly sensitive to local structural perturbations [32]. Taken together, these observations suggest that deletions and amino acid substitutions in loop 2 of RpsE are predicted to alter the local structure and dynamics of the h34 region, which constitutes the spectinomycin-sensitive contact zone. Such changes disrupt the configuration of this structural node and weaken the ability of spectinomycin to stabilize the partially rotated conformation of the 30S head required for inhibition of translocation, which may contribute to a sharp increase in MIC.

The RpsE-dependent pathway, which dominated under our in vitro conditions, is not the only possible route to spectinomycin resistance. In natural populations of *Neisseria* and other bacteria, resistance has also been reported to arise through substitutions in the 16S rRNA (e.g., G1064C, C1192T), located within the spectinomycin-binding region of h34, which likewise confer high-level resistance to the antibiotic [34,38]. Such variants likely did not emerge in our experiments because multiple mutations would be required across several rRNA operons, and because these changes may impose higher growth costs that demand a specific genomic context for compensation. Thus, mutations in RpsE represent the fastest and most accessible route under laboratory selection, but they do not preclude the existence of alternative evolutionary trajectories in nature.

In the highly resistant *N. gonorrhoeae* Clinical line “D”, we identified a triple deletion, ΔArg31–Met33, in loop 2 of RpsE, combined with a Gly30Val substitution. A similar deletion (ΔMet31–Phe33) in the homologous region of RpsE was previously observed during spectinomycin selection in *Pasteurella multocida* [45] and was likewise associated with a high level of resistance (MIC = 512 mg/L). In *N. lactamica* line “O”, we detected a triple deletion, ΔVal27–Gly29, in the same domain. A mutation with a closely related localization, ΔVal27 combined with Lys28Glu, which produces a comparable phenotype (MIC > 1024 mg/L), has been reported in clinical isolates of *N. gonorrhoeae* from Norway [35] and China [43].

Notably, the *N. sicca* lines “T”, “U”, and “V” (MIC > 2048 mg/L) acquired the Thr24Pro mutation in RpsE during in vitro selection, a substitution previously reported in clinical *N. gonorrhoeae* isolates [35,48]. In those studies, this mutation was associated with only low-level resistance (MIC = 128 mg/L), rather than the high-level resistance observed in our experiments. The additional contribution of the accompanying Met9Val substitution in RpsB is likely, although its independent role remains unclear.

The *N. gonorrhoeae* Clinical line “C” acquired a Trp23Cys mutation in RpsB (ribosomal protein S2), which resulted in low-level resistance (MIC = 128 mg/L). Determinants of spectinomycin resistance in this gene have not previously been reported in *N. gonorrhoeae*. However, data from *Bacillus subtilis*, where in vitro selection yields substitutions in the homologous region (Pro22Arg and Pro25Leu) [37], suggest that this segment of S2 represents a mutation-sensitive element. Given that S2 is part of the network of ribosomal proteins coordinating platform and head movements of the 30S subunit [33], and that spectinomycin disrupts the coordination of these motions [32], it is plausible that mutations in RpsB indirectly alter the dynamics of the small subunit and thereby reduce drug sensitivity without affecting the antibiotic’s direct interaction with h34.

In a recent study by Li et al. [42] selection of the *N. gonorrhoeae* WHO G strain yielded a ΔGly30 deletion in RpsE, which resulted in high-level resistance (MIC = 2048 mg/L) accompanied by pronounced growth costs. Our findings are consistent with these observations. In contrast to [42], we observed a broad spectrum of independent deletion variants arising across different *Neisseria* species, yet consistently converging on loop 2 of RpsE. Together, these observations indicate that this region represents a constrained mutational pathway. In the strains and species examined here, it appears to be a recurrent and strongly favored route to high-level spectinomycin resistance. Although the present experiments do not constitute isogenic reconstruction of individual *rpsE* alleles, the repeated association of high-level resistance with loop 2 alterations in two *N. gonorrhoeae* backgrounds and in *N. lactamica*, together with published clinical and experimental observations [35,42,43], supports a constrained mutational landscape. For lines carrying additional mutations, the contribution of accompanying changes cannot be formally excluded; these cases are therefore described as associations rather than definitive proof of causality.

Mutations were also identified in the Rne/Rng-family ribonuclease (RNase E, according to PubMLST), which independently acquired mutations during selection in several samples (Ala304Thr in *N. gonorrhoeae* Clinical line “F”; Gly61Glu in *N. gonorrhoeae* ATCC lines “K”, “L”, “M”, and “N”). This enzyme is involved in rRNA and tRNA maturation as well as mRNA degradation, and may therefore influence the expression of hundreds of cellular proteins. We hypothesize that the mutations identified in RNase E may have altered the expression levels of ribosomal proteins and 16S rRNA, indirectly affecting spectinomycin susceptibility. In addition to Ala304Thr in the Rne/Rng-family ribonuclease, the *N. gonorrhoeae* Clinical line “F” (MIC = 128 mg/L) carried an Asp259Gly substitution in the lytic murein transglycosylase MltG, which participates in peptidoglycan biosynthesis. Mutations in another lytic murein transglycosylase, MltB, have previously been reported to influence spectinomycin resistance in *Lysobacter enzymogenes* [49], supporting a potential role for cell-wall-remodeling genes in shaping resistance phenotypes.

Finally, mutations were also identified in the lipid A export permease/ATP-binding protein MsbA. In the *N. gonorrhoeae* ATCC line “N” (MIC = 128 mg/L), we detected an Asp175Asn substitution, whereas in the *N. sicca* line “V” (MIC > 2048 mg/L) we observed an Ala44Ser substitution. MsbA is an ABC transporter responsible for flipping lipid A across the inner membrane [50] but it also mediates the efflux of various xenobiotics. Although spectinomycin is not a typical substrate of ABC transporters, alterations in MsbA activity may influence overall membrane permeability or act synergistically with other mutations.

### 3.2. Growth-Rate and Structural Effects of Observed Mutations

Analysis of growth curves shows that substitutions in loop 2 of S5, which are associated with the highest increases in MIC, are accompanied by a marked reduction in the growth rate parameter *r*. The heatmap of contact-energy changes (Figure 6) suggests that the magnitude of growth costs appears to be associated not with the overall stability of the S5-environment interface (GBVI and TCE values in all mutants remain close to WT), but rather with the emergence of new strongly destabilizing or stabilizing contacts.

For line “G” (ΔIle32/ΔIle30 in *E. coli*, *r* = 0.79), the data show that extensive structural rearrangements do not necessarily lead to the most severe loss of viability. In this mutant, Lys28 (Lys26) switches from A923 to C1069 (−25.9 kcal/mol), the Lys25(Lys23)–G922 interaction is retained (−17.4 kcal/mol), and strong new contacts appear: Arg31(Arg29)–C1071 (−27.2 kcal/mol) and Arg31(Arg29)–G1193 (−18.3 kcal/mol).

The most pronounced reduction in growth rate is observed in line “J” (ΔVal23–Thr24/ΔVal21–Thr22, *r* = 0.58), which exhibits a profile in which the Lys28(Lys26)–A923 interaction becomes strongly strengthened (−33.0 kcal/mol) and the Lys25(Lys23)–G922 contact is completely lost, whereas Arg22(Arg20)–U17 is sharply strengthened (from −6.6 to −27.9 kcal/mol). New contacts appear, including Gly29(Gly27)–U1070 (−6.3 kcal/mol) and Thr26(Thr24)–A1081 (−6.5 kcal/mol), which are absent in other mutants, and the interaction energy of Lys25(Lys23)–A1082 increases from −18.4 to −38.4 kcal/mol.

Distinctive features of line “D” (Gly30Val + ΔArg31–Met33, *r* = 0.79) included the switching of the Lys28 (Lys26) contact from A923 to U1070 (from 0 to −21.2 kcal/mol), rather than to C1069, as well as a marked strengthening of the Lys28(Lys26)–spectinomycin interaction (from +1.2 to −13.0 kcal/mol) and pronounced destabilization of the Lys54(Lys52)–A1080 contact (ΔE = +18.3 kcal/mol). As in line “J”, new protein–protein contacts emerged (e.g., Glu67(Glu65)–S2:Lys104, −21.4 kcal/mol). These observations indicate that the specific pattern of altered contacts, rather than aggregate energetic metrics, determines the growth cost of resistance.

In our experiments, spectinomycin resistance in the examined *N. gonorrhoeae* and commensal *Neisseria* strains followed a unified trajectory. An initial latent phase was observed, during which the population accumulated genetic diversity. This was followed by an abrupt increase in MIC above 2048 mg/L, driven by a single mutation in loop 2 of S5. Continued cultivation led to the emergence of compensatory changes that mitigated the physiological consequences of the primary resistance mutation. Some of these secondary mutations were located in other proteins of the small ribosomal subunit (RpsB, RpsC, RpsK, RpsU). Restoration of growth rate through secondary mutations in RpsB, RpsC, and other small-subunit proteins may reflect their involvement in the same network of dynamic interactions that ensures coordinated movement of ribosomal components [33].

At the molecular level, compensatory tendencies manifest in the emergence of new stabilizing contacts between S5 and proteins S8 and S2, observed in all mutants (Lys161(Lys159)–Lys63 of S8: from 0 to −15.3 kcal/mol; Gly160(Gly158)–Lys63 of S8: from 0 to −11.4 kcal/mol; and, in lineages “D” and “J”, Glu67(Glu65)–Lys104 of S2: from 0 to −21.4 kcal/mol). These interactions likely partially compensate for the loss of the native geometry of loop 2.

Many mutations in loop 2 of RpsE, as shown in [35], disrupt ribosome assembly and reduce translational accuracy. Such defects in translation likely caused the critical loss of viability in several potentially resistant variants, excluding them from further evolutionary trajectories (Figure 1, “unviable cell lines”).

Interestingly, Jena et al. [46] described two qualitatively distinct adaptive pathways when *E. coli* was selected on spectinomycin-containing medium: a classical resistance route (mutations in RpsE that increase MIC) and a noncanonical adaptation route, in which bacteria acquired mutations in other genes and improved fitness in the presence of the antibiotic without altering MIC. The authors attribute noncanonical adaptation to mutations in alternative target genes distinct from *rpsE*, or in entirely novel loci such as the multidrug transporter *mdfA*. It is possible that the cell lines we observed with MIC = 128 mg/L and mutations in *rpsB*, *rne*, *mltG*, and *msbA* represent an analogous strategy of adaptation to low antibiotic doses, rather than merely an “intermediate” step toward high-level resistance. As Jena et al. [46] correctly note, relying solely on MIC may fail to capture the full spectrum of bacterial adaptive strategies, particularly under sublethal antibiotic concentrations. In this context, our analysis of growth curves and the parameter *r* allowed us to detect differences in viability between cell lines with identical MIC values but distinct mutations in RpsE.

Additional data obtained by Nabu et al. [40] extend this picture at the proteomic level. Examining a clinical *N. gonorrhoeae* strain with high-level resistance (MIC > 1024 mg/L), the authors showed that, despite an overall ≈96% similarity of proteomic profiles to the susceptible strain, the resistant variant exhibited altered expression of a limited set of proteins, including translation factors and components of energy metabolism. Under sub-inhibitory concentrations of spectinomycin, both strains displayed a dose-dependent induction of the 50S ribosomal protein L7/L12 and enzymes of central metabolism, which was interpreted as a compensatory response to protein synthesis inhibition.

Taken together, our findings and those of Nabu et al. [40] suggest that high-level spectinomycin resistance in *N. gonorrhoeae* involves not only localized disruption of the RpsE-16S rRNA interface but also broader remodeling of the ribosomal apparatus and energy metabolism, which may contribute to partial compensation for growth costs.

### 3.3. Lack of Cross-Resistance with Aminoglycosides

Although in some cases the identified mutations were accompanied by a 2–3-fold increase in kanamycin and gentamicin MICs, the clinical resistance threshold was not exceeded (Table 2). The absence of pronounced cross-resistance can be explained by the distinct binding sites of these antibiotics: aminoglycosides (first-generation kanamycin and second-generation gentamicin) interact with the 16S rRNA within the decoding center of the A-site, whereas spectinomycin binds to 16S rRNA near helix 34. This functional separation is supported by the findings of Golparian et al. [45], who showed that selection for gentamicin resistance resulted in a proportional increase in kanamycin MIC, while spectinomycin MIC remained unchanged. The mutations in that study were located in *fusA* (elongation factor EF-G) and *ubiM* (ubiquinone biosynthesis), i.e., in genes unrelated to the structural spectinomycin-binding site in h34 of 16S rRNA. It should be noted that our study examined only a limited set of aminoglycosides (kanamycin and gentamicin), and other members of this class may theoretically display different susceptibility profiles. However, additional evidence indicates that other aminoglycosides also retain activity against spectinomycin-resistant strains. In particular, apramycinthat also binds to the ribosomal A-site, exhibits low MICs (16–32 mg/L) against *N. gonorrhoeae* strains carrying both the C1192T mutation in 16S rRNA and the Thr24Pro substitution in RpsE [51].

Thus, even high-level spectinomycin resistance was not accompanied by a substantial reduction in susceptibility to aminoglycosides, underscoring the independence of their mechanisms of action and resistance. However, this interpretation should be considered cautiously because the thresholds used here are provisional literature-based criteria rather than formally endorsed the European Committee on Antimicrobial Susceptibility Testing (EUCAST) or the Clinical and Laboratory Standards Institute (CLSI) clinical breakpoints for *Neisseria*.

### 3.4. Evolutionary and Clinical Implications

The data we obtained allow a revised interpretation of the historical dynamics of spectinomycin resistance in *N. gonorrhoeae*. The narrow evolutionary corridor leading to high-level resistance through mutations in loop 2 of RpsE is likely one of the few viable routes to achieving such resistance levels. This form of resistance has been shown to incur a cost in the form of reduced growth rate. According to current understanding, this cost arises from disruption of the coordinated dynamics of the 30S subunit [32,33]. As a result, resistant variants may emerge under strong selective pressure but are outcompeted by more fit strains in the absence of the antibiotic. This explains why, after spectinomycin use was discontinued, the frequency of resistant strains declined relatively rapidly, unlike the situations with penicillins, tetracyclines, or fluoroquinolones, where multiple independent resistance pathways and low mutation costs promote long-term fixation of resistant genotypes in the population.

Rapid increases in resistance during periods of spectinomycin use, followed by its relatively rapid decline after the drug was discontinued, have been documented across multiple countries and time periods. In the Philippines, after a surge in resistance and subsequent withdrawal of the drug, the proportion of resistant isolates in a cohort of 140 individuals decreased from 22% to below 10% within less than two years (1988–1989) [21,22]. In Thailand, resistant strains were no longer detected only five years after the outbreak of 1994–1995 [23]. In South Korea, where the prevalence of resistant strains reached 11% in a sample of 16,000 individuals by 1985, no resistant isolates have been identified since 2002 [24]. In England, following the London outbreak of the 1980s, resistant isolates have been reported only sporadically, with just six cases recorded between 2000 and 2016 [26]. In China, 1.3% resistant and 4% intermediate isolates were reported in 1987–1992, but they have since been observed only rarely [52]. Notably, this pattern persists despite the relatively frequent use of spectinomycin in China (prescribed by 7.9% of 1890 surveyed physicians [53]) against the backdrop of widespread ceftriaxone resistance [54,55]. These observations provide indirect support for the growth costs incurred by *N. gonorrhoeae* during adaptation to spectinomycin.

The pharmacological properties of spectinomycin are also clinically relevant. Its distinct chemical structure is associated with the absence of the pronounced nephrotoxicity and ototoxicity characteristic of aminoglycosides, as well as with the lack of marked cross-resistance to them. These features make spectinomycin a potentially useful therapeutic option, particularly in the context of rising resistance to cephalosporins and macrolides. However, the identification of compensatory mutations in *rpsB*, *rpsC*, *rpsK*, and *rpsU* that partially restore the viability of highly resistant variants (Section 3.2) serves as an important caution. Despite their reduced growth rates, such strains remain viable. With prolonged and widespread use of spectinomycin, the gradual accumulation of compensatory changes that diminish growth costs (and thereby increase the likelihood of resistant clones becoming established in the population) cannot be excluded. Importantly, these in vitro growth measurements do not directly test transmission, within-host persistence, or population-level dynamics; therefore, clinical extrapolation requires caution. This underscores the need for continuous genomic surveillance and a cautious approach to any potential expansion of the clinical use of spectinomycin.

From a diagnostic perspective, our data suggest that the most informative target for a molecular test of high-level spectinomycin resistance is loop 2 of RpsE (codons 19–33). Due to the broad distribution of resistance-associated substitutions and deletions throughout this loop, a sequencing-based assay covering the entire loop 2 region would be required, rather than a probe targeting an individual position. The classical 16S rRNA determinants (G1064C and C1192T, *E. coli* numbering) should be included as additional targets. By contrast, the low-level resistance determinants identified here (in *rpsB*, RNase E, *mltG*, and *msbA*) are unsuitable as stand-alone diagnostic markers.

### 3.5. Limitations

Several limitations should be considered when interpreting these results. First, the number of parental genetic backgrounds and independent selection series was limited, and broader panels of clinical isolates will be needed to test how general this mutational pattern is. Second, individual *rpsE* alleles and candidate compensatory mutations were not reconstructed in isogenic backgrounds; therefore, causality is inferred from convergence, genomic simplicity in selected lineages, and published observations rather than directly demonstrated. Third, growth-curve measurements provide an in vitro proxy for one component of fitness and do not directly test competition, transmission, within-host persistence, or population-level dynamics; growth data were not obtained for the *N. sicca* derivatives. Fourth, the disc-based selection protocol is a specific in vitro enrichment approach based on a spatial spectinomycin gradient and may favour particular accessible mutational routes. Similar RpsE loop 2 alterations have also been reported in the literature, suggesting that the observed convergence is not solely an artefact of the disc-based method. Finally, interpretation of gentamicin and kanamycin cross-resistance relies on provisional thresholds proposed in the literature rather than formally endorsed *Neisseria*-specific EUCAST or CLSI breakpoints.

## 4. Materials and Methods

### 4.1. Bacterial Strains

Four *Neisseria* strains were used in this study: a clinical isolate of *N. gonorrhoeae* susceptible to all tested antimicrobials (MICs: PEN = 0.12 mg/L, TET = 0.06 mg/L, CRO = 0.004 mg/L, CIP = 0.004 mg/L, SPC = 16 mg/L, AZT = 0.06 mg/L, KAN = 4 mg/L, GEN = 4 mg/L), obtained from a urogenital swab of a patient in Russia in 2017 as part of routine antimicrobial-resistance surveillance (strain 10269, MLST 14013; complete genome under accession GCA_025169995.2 [56]); the reference strain *N. gonorrhoeae* ATCC 49226 (PEN = 0.5 mg/L, TET = 0.5 mg/L, CRO = 0.008 mg/L, CIP = 0.008 mg/L, SPC = 32 mg/L, AZT = 0.5 mg/L, KAN = 16 mg/L, GEN = 4 mg/L, MLST 11075); the reference strain *N. lactamica* ATCC 23970 (SPC = 32 mg/L, KAN = 16 mg/L, GEN = 4 mg/L); and the reference strain *N. sicca* ATCC 9913 (SPC = 16 mg/L, KAN = 16 mg/L, GEN = 8 mg/L). All reference strains were supplied as Culti-Loops (Thermo Scientific, Waltham, MA, USA).

### 4.2. Selection of Spectinomycin-Resistant Mutants

Filter paper discs (5 mm in diameter) were prepared and each was impregnated with 5 µL of a sterile solution of spectinomycin dihydrochloride pentahydrate at a concentration of 20 mg/mL (Central Drug House Ltd., Mumbai, India). Initial cultures of *Neisseria* resuspended in phosphate-buffered saline (PBS) (diaGene, Moscow, Russia) to a density of 0.5 McFarland were inoculated onto ready-to-use chocolate agar plates (Hem Ltd., Moscow, Russia). Two to three spectinomycin-containing discs were then placed onto the agar surface, and the plates were incubated for 24–48 h at 37 °C in 5% CO_2_. The radius of growth inhibition was observed, and colonies appearing at the edge of the inhibition zone were collected, resuspended, and subjected to the same procedure. After every ten passages, or upon a marked reduction in the inhibition radius, a pure culture was resuspended in 100 µL of sterile lyophilization buffer (70 mL MilliQ water, 30 mL of a 7.5% albumin solution, and 5.0 g of myo-inositol; PanEco, Moscow, Russia) to a density of 2.0 McFarland and lyophilized for long-term storage. The schematic trajectories shown in Figure 1 and Figure 2 include both independent selection series initiated from separate colonies and sequential derivatives obtained by continued passaging within a lineage.

### 4.3. Antimicrobial Susceptibility Testing

Susceptibility to antimicrobial agents was determined by the agar dilution method using a solid medium (Himedia GC Agar Base Kit, Mumbai, India) supplemented with 4–2048 mg/L spectinomycin. Susceptibility to kanamycin sulfate and gentamicin sulfate was assessed in the same manner (1–512 mg/L; Central Drug House Ltd., Mumbai, India). The reference strain *N. gonorrhoeae* ATCC 49226 was used as a control. Resistance breakpoints were set at >64 mg/L for spectinomycin (according to EUCAST v.16.0), >16 mg/L for gentamicin (as proposed in [45]) and >64 mg/L for kanamycin (as proposed in [44]). Gentamicin and kanamycin MICs were interpreted using provisional thresholds as EUCAST and CLSI do not currently provide formally endorsed *Neisseria*-specific clinical breakpoints for these drugs.

### 4.4. Whole-Genome Sequencing, Read Mapping, and Variant Calling

Genomic DNA from all *Neisseria* cell lines was extracted using the Monarch Genomic DNA Purification Kit (New England Biolabs, Ipswich, MA, USA) according to the manufacturer’s instructions. Genomic DNA concentrations were measured using a Qubit fluorometer with the dsDNA HS Assay Kit (Invitrogen, Carlsbad, CA, USA). Library quality was assessed on an Agilent TapeStation 4150 using the High Sensitivity D1000 kit (Agilent Technologies, Santa Clara, CA, USA). Paired-end sequencing was performed on the DNBSEQ G-400 platform using the DNBSEQ-G400RS High-throughput Sequencing Set PE150, following the manufacturer’s protocol (MGI Tech, Shenzhen, China). FASTQ files were generated using the zebracall software v2.0 (MGI Tech, Shenzhen, China). Raw read quality was assessed using FastQC v0.12.1 [57]. Adapter removal and quality trimming were performed with fastp v1.0.1 [58] using the following parameters: trimming 9 nucleotides from each end of the read, filtering out reads shorter than 50 bp or with an average quality score below Q20, and applying a sliding window of 8 nucleotides. Processed reads were mapped to the corresponding ATCC reference genome (https://genomes.atcc.org, accessed 1 September 2025) using bwa-mem2 v2.3 [59]. For the *N. gonorrhoeae* Clinical isolate, the complete genome GCA_025169995.2, previously sequenced by our group [56], was used as the reference. Also, the parental *N. lactamica* cell line shown in Figure 2 was not included in the genomic analysis because the corresponding library failed sequencing quality control. As a reference, we used the ATCC 23970 genome from the ATCC official website. SAM files were converted to BAM format and sorted using samtools v1.10 [60], followed by variant calling with freebayes v1.3.10 [61]. Variants were filtered with bcftools v1.22 [60] using the following thresholds: minimum quality 20, minimum depth 10×, and a ratio of reads supporting the variant to total depth of at least 0.9. All variants were additionally inspected manually in alignments using Ugene v52.0 [62]. Finally, genomic annotation of variants was performed using SnpEff v5.2 [63] based on the corresponding reference .gbk files. Raw sequencing reads generated in this study have been deposited in BioProject PRJNA1447786.

### 4.5. Growth Kinetics Assay (CFU-Based)

The lyophilized cell line was rehydrated in 200 µL of PBS and grown on chocolate agar strictly for 24 h to obtain a culture in the late logarithmic phase. Colonies were resuspended in 2 mL of PBS to a density of 0.5 McFarland (10^8^ CFU/mL), diluted 10^5^-fold, and inoculated at 100 µL onto a series of plates. After the designated incubation time (from 2 to 48 h), colonies were washed from the plates with 500 µL of PBS, and 50 µL of the resulting suspension were plated onto fresh agar in triplicate. Starting from the 8-h time point, the suspension was additionally diluted (with the dilution factor increasing from 10^1^ up to 10^7^ as the culture grew) to prevent lawn formation. After 24 h of incubation, plates were photographed, colonies were counted visually, and the counts were multiplied by the corresponding dilution factor to obtain an approximate CFU/mL value.

### 4.6. Estimation of Logistic Growth Parameters

The obtained CFU (colony-forming units per plate) data were analyzed in Python 3.12.2 using pandas 2.3.3 and NumPy 2.2.5. Data preprocessing relied on standard pandas operations (read_csv, merge, groupby, agg, DataFrame.apply, to_numpy) for parsing CFU tables and aligning replicate measurements. NumPy routines (log, exp, array, and random-sampling functions) were used for computing log-residuals, evaluating the logistic model, and performing bootstrap resampling. To characterize the growth curves, parameters of the logistic model were estimated: *dN*/*dt* = *rN*(*1* − *N*/*N_∞_*), with the analytical solution *N*(*t*) = *N_∞_*/[1 + ((*N_∞_* − *N*_0_)/*N*_0_) * *exp*(−*r*(*t* − *t*_0_))]. In this phenomenological model, *N* denotes the CFU count, t the time in hours, *r* the growth rate, and *N_∞_* the carrying capacity. The parameters *r* and *N_∞_* were estimated by maximum likelihood under the assumption of log-normal errors. The sum of squared log-residuals was minimized. Parameter uncertainty was assessed in two ways: asymptotically, using the Fisher information matrix for the log-parameters (with regularization applied when the condition number exceeded 10^12^), and by bootstrapping biological replicates (1000 iterations, percentile-based 95% confidence intervals). Goodness of fit was evaluated using R^2^ (in both log and linear scales), RMSE(log), AIC, and BIC; normality of residuals was assessed with the Shapiro–Wilk test. No proprietary software was used; all computations were performed with standard Python libraries using the parameters described above.

### 4.7. Molecular Modeling and Interaction Energy Calculations

Molecular modeling and all energy calculations were performed in the MOE software package, v2019.0102 (Molecular Operating Environment; Chemical Computing Group, Montreal, QC, Canada) using the Amber10:EHT force field and the Generalized Born/Volume Integral (GB/VI) implicit solvation model. The GB/VI model estimates the electrostatic component of the solvation free energy using a Generalized Born formalism, in which effective Born radii are obtained from a volume-integral approximation of atomic solvent accessibility. A non-polar term proportional to the solvent-accessible surface area is also included. Together, these contributions yield the GBVI solvation energy (kcal/mol), which reflects the combined polar and hydrophobic stabilization of the structure in an implicit aqueous environment. In this study, GBVI values were used as an integral descriptor of the stability of the S5 protein across all wild-type and mutant models. The crystal structure of the *Escherichia coli* 30S ribosomal subunit in complex with spectinomycin (PDB ID: 4V56, 3.93 Å resolution) was used as the template. From this structure, the ribosomal protein S5 chain (chain AE, residues 1–158) was extracted. The amino acid sequence of *E. coli* S5 is homologous to that of *Neisseria* (*N. gonorrhoeae*, *N. lactamica*, *N. sicca*) in the loop 2 region (residues 20–31 according to *E. coli* numbering), allowing direct comparison. Based on the wild-type (WT) structure, seven mutant models corresponding to the variants identified experimentally were constructed (residue numbering as defined in Section 2.4). Mutant structures were generated by homology modeling while preserving the structural environment of S5 within the ribosome. The positions of all other components of the 30S subunit (16S rRNA, proteins S2, S3, S4, and S8, Mg^2+^ ions, water molecules, and spectinomycin) were kept unchanged. Each model was subjected to energy minimization in the Amber10:EHT force field with GB/VI until the root-mean-square gradient reached 0.01 kcal·mol^−1^·Å^−1^. For all minimized structures, GBVI solvation energies were computed as described above. Pairwise interaction energies between S5 and its molecular environment were calculated using the Protein → Contacts function in MOE. Set A included all atoms of S5, and Set B included all atoms of the remaining components of the system. For each residue pair, the program computed the total non-bonded interaction energy (Energy, kcal/mol); positive values correspond to destabilizing interactions, whereas negative values correspond to stabilizing interactions. To obtain an integrated descriptor of the S5-environment interface, all Energy values from the contact analysis for a given model were summed. This quantity was defined as the total contact energy (TCE). Higher positive TCE values indicate stronger destabilization of the interface. Complete contact tables for the wild-type model and seven mutant variants are provided in Supplementary Table S2. GBVI and TCE values for all models, along with analysis of key contacts, are presented in Section 2.

## 5. Conclusions

The in vitro selection of four *Neisseria* strains provided evidence that the emergence of high-level spectinomycin resistance follows a narrow and reproducible mutational trajectory. Across the independent selection series examined here, high-level resistance was consistently associated with mutations in loop 2 of RpsE. Molecular modelling suggested that these mutations disrupt the balanced network of stabilizing contacts between S5 residues Lys25 (Lys23 in *E. coli* numbering) and Lys28 (Lys26 in *E. coli*) as well as helix 34 of 16S rRNA (G922, A923, C1069), potentially impairing the drug-binding site. In the cell lines analysed by growth-curve modelling, loop 2 mutations were associated with measurable reductions in growth performance, reflected in a decreased growth rate. The magnitude of the growth deficit was associated with changes in specific local contact energies rather than with the overall stability of the S5–ribosome interface. Prolonged selection resulted in compensatory mutations in other ribosomal proteins (RpsB, RpsC, RpsK, RpsU) and metabolic regulators, which partially restored growth without eliminating the growth burden. Notably, no clinically relevant cross-resistance to gentamicin or kanamycin was detected under the provisional thresholds used here. The convergence of resistance mutations to a single hotspot in RpsE may help explain the rapid rise and subsequent decline of spectinomycin resistance in natural populations. Under drug pressure, high-level resistance emerges readily, but the associated growth costs may contribute to the decline of resistant clones once the drug is withdrawn. However, these growth costs represent only one factor that may influence the persistence of resistant strains. Clinical outcomes also depend on transmission, within-host persistence, treatment practices, antimicrobial exposure, and population-level dynamics, which were not directly addressed in this study. These findings are consistent with the potential clinical utility of spectinomycin while underscoring the importance of genomic surveillance.

## Figures and Tables

**Table 1:** Summary of verified mutations in strains obtained through stepwise selection. Putative resistance markers are shown in bold. S—susceptible, R—resistant, HLR—high-level resistant.

Strain	Line ID	SPC MIC, mg/L	Non-Synonymous Mutations (Relative to Reference)
*N. gonorrhoeae* Clinical	A	16 (S)	Parental *
*N. gonorrhoeae* Clinical	B	16 (S)	-
*N. gonorrhoeae* Clinical	C	128 (R)	**Trp23Cys (RpsB)**; Gly15Asp (Ppx/GppA phosphatase family protein); Ala165fs (site-specific DNA-methyltransferase); Asn61His (pilin)
*N. gonorrhoeae* Clinical	D	>2048 (HLR)	**Gly30Val + ΔArg31-Met33 (RpsE)**
*N. gonorrhoeae* Clinical	E	16 (S)	Ser21fs (opacity family porin); Lys65Glu, Asp66Asn & Gly69Thr (pilin)
*N. gonorrhoeae* Clinical	F	128 (R)	**Ala304Thr (Rne/Rng family ribonuclease)**; Ser21fs (opacity family porin); **Asp259Gly (endolytic transglycosylase MltG)**
*N. gonorrhoeae* Clinical	G	>2048 (HLR)	**ΔIle32 (RpsE)**
*N. gonorrhoeae* ATCC 49226	H	32 (S)	Parental *
*N. gonorrhoeae* ATCC 49226	I	32 (S)	Gly55Asp (cold-shock protein); Ala35fs (sodium–proton antiporter)
*N. gonorrhoeae* ATCC 49226	J	>2048 (HLR)	Gly55Asp (cold-shock protein); Ala35fs (sodium–proton antiporter); **ΔVal23-Thr24 (RpsE)**; **ins56GlnLysArgLeu (RpsU)**
*N. gonorrhoeae* ATCC 49226	K	32 (S)	Gly55Asp (cold-shock protein); Ala35fs (sodium–proton antiporter); Gly61Glu (Rne/Rng family ribonuclease)
*N. gonorrhoeae* ATCC 49226	L	32 (S)	Gly55Asp (cold-shock protein); Ala35fs (sodium–proton antiporter); Gly61Glu (Rne/Rng family ribonuclease); Ala27fs (opacity family porin); intergenic C1486410T (NCS2 family permease)–(cell division protein FtsZ)
*N. gonorrhoeae* ATCC 49226	M	32 (S)	Gly55Asp (cold-shock protein); Ala35fs (sodium–proton antiporter); Gly61Glu (Rne/Rng family ribonuclease); Ala27fs (opacity family porin); intergenic C1486410T (NCS2 family permease)–(cell division protein FtsZ); Gln118fs (MtrC); Thr361Met (zinc metalloprotease FtsH); intergenic ins1399438AAGC (chromosome segregation protein Smc)–(hypothetical protein)
*N. gonorrhoeae* ATCC 49226	N	128 (R)	Gly55Asp (cold-shock protein); Ala35fs (sodium–proton antiporter); Gly61Glu (Rne/Rng family ribonuclease); Ala27fs (opacity family porin); intergenic C1486410T (NCS2 family permease)–(cell division protein FtsZ); **Asp175Asn (lipid A export permease/ATP-binding protein MsbA)**
*N. lactamica* ATCC 23970	not applicable	32 (S)	Parental *
*N. lactamica* ATCC 23970	O	>2048 (HLR)	**ΔVal27-Gly29 (RpsE)**
*N. lactamica* ATCC 23970	P	>2048 (HLR)	**ΔVal27-Gly29 (RpsE)**; **Trp167Arg (RpsC)**; intergenic G1391436A (gdhA, NADP-specific glutamate dehydrogenase)–(pdhR, Pyruvate dehydrogenase complex repressor)
*N. lactamica* ATCC 23970	Q	>2048 (HLR)	**Val23Met (RpsE)**; **Ser97Phe (RpsK)**; Glu66Lys (hypothetical protein); Ser259Gly (Tn7 transposition protein TnsB); Ser483Pro (Sodium/proline symporter PutP); intergenic C877962T (Cell division protein FtsB)–(Ribonucleoside-diphosphate reductase NrdB); Lys179fs (hypothetical protein); Glu198Lys (UDP-3-O-acyl-N-acetylglucosamine deacetylase LpxC)
*N. lactamica* ATCC 23970	R	>2048 (HLR)	**Val23Met (RpsE)**; **Gly30Ser (RpsE)**; Cys66Tyr (NADP transhydrogenase PntA); Pro70Ser (DUF2061 domain-containing protein); intergenic 1670232delAGAAG (pilin PilC)–(Bifunctional riboflavin kinase RibF); intergenic 2110782delT (groL, chaperonin GroEL)–(sodium-dependent transporter)
*N. sicca* ATCC 9913	S	16 (S)	Parental *
*N. sicca* ATCC 9913	T	>2048 (HLR)	**Thr24Pro (RpsE)**; **Met9Val (RpsB)**; Thr183Asn (Translocation and assembly module subunit TamA)
*N. sicca* ATCC 9913	U	>2048 (HLR)	**Thr24Pro (RpsE)**; **Met9Val (RpsB)**; Met5fs (hypothetical protein); Val490fs (hypothetical protein); Gly176fs (hypothetical protein); Gly234Val (hypothetical protein); Glu198Asp (NAD(+) synthetase NadE); Ala269Ser (N-acetylmuramoyl-L-alanyl-D-glutamate ligase MurE); Ala215Ser (Inner membrane protein YccS); intergenic C136005A (chromosome-partitioning protein ParB)–(ATP synthase AtpB); intergenic C1208610T (Aspartokinase LysC)–(Molybdenum-pterin-binding protein MopA); intergenic G546566A (hypothetical protein)–(sulfite reductase flavoprotein CysJ); intergenic T1966430C (hypothetical protein)–(hypothetical protein); intergenic 776211delTTTCAGACGACCTTT (hypothetical protein)–(C4-dicarboxylate transporter DcuB)
*N. sicca* ATCC 9913	V	>2048 (HLR)	**Thr24Pro (RpsE)**; **Met9Val (RpsB)**; Ala44Ser (lipid A export permease/ATP-binding protein MsbA); Gly147Cys (Ribosome biogenesis GTPase RbgA); plus multiple mutations in intergenic regions and genes encoding hypothetical proteins (full list in Supplementary Table S1)

* For parental lines (A, H, etc.), “Parental” indicates the reference genome used for variant calling.

**Table 2:** Susceptibility of parental and derived *Neisseria* cell lines to spectinomycin (SPC), kanamycin (KAN), and gentamicin (GEN) (mg/L). S—susceptible, R—resistant, HLR—high-level resistant.

Strain	Line ID	MIC, mg/L		
		SPC	KAN	GEN
*N. gonorrhoeae* Clinical	A	16 (S)	4 (S)	4 (S)
*N. gonorrhoeae* Clinical	B	16 (S)	4–8 (S)	4 (S)
*N. gonorrhoeae* Clinical	C	128 (R)	8 (S)	4 (S)
*N. gonorrhoeae* Clinical	D	>2048 (HLR)	32 (S)	4–8 (S)
*N. gonorrhoeae* Clinical	E	16 (S)	4 (S)	4–8 (S)
*N. gonorrhoeae* Clinical	F	128 (R)	4–8 (S)	4–8 (S)
*N. gonorrhoeae* Clinical	G	>2048 (HLR)	8 (S)	4 (S)
*N. gonorrhoeae* ATCC 49226	H	32 (S)	16 (S)	4–8 (S)
*N. gonorrhoeae* ATCC 49226	I	32 (S)	16–32 (S)	8–16 (S)
*N. gonorrhoeae* ATCC 49226	J	>2048 (HLR)	32–64 (S)	16 (S)
*N. gonorrhoeae* ATCC 49226	K	32 (S)	16 (S)	4–8 (S)
*N. gonorrhoeae* ATCC 49226	L	32 (S)	16–32 (S)	4–8 (S)
*N. gonorrhoeae* ATCC 49226	M	32 (S)	16 (S)	8–16 (S)
*N. gonorrhoeae* ATCC 49226	N	128 (R)	16	4–8 (S)
*N. lactamica* ATCC 23970	not applicable	32 (S)	8–16 (S)	4–8 (S)
*N. lactamica* ATCC 23970	O	>2048 (HLR)	16 (S)	8 (S)
*N. lactamica* ATCC 23970	P	>2048 (HLR)	16–32 (S)	16 (S)
*N. lactamica* ATCC 23970	Q	>2048 (HLR)	8–16 (S)	4 (S)
*N. lactamica* ATCC 23970	R	>2048 (HLR)	8 (S)	4 (S)
*N. sicca* ATCC 9913	S	16 (S)	16–32 (S)	8–16 (S)
*N. sicca* ATCC 9913	T	>2048 (HLR)	32–64 (S)	8–16 (S)
*N. sicca* ATCC 9913	U	>2048 (HLR)	32–64 (S)	16 (S)
*N. sicca* ATCC 9913	V	>2048 (HLR)	32–64 (S)	16 (S)

**Table 3:** Solvation free energy (GBVI) and total contact energy (TCE) of wild-type and mutant RpsE variants in complex with the ribosome.

RpsE Mutations (*N. gonorrhoeae* Numbering)	RpsE Mutations (*E. coli* Numbering)	GBVI, kcal/mol	TCE, kcal/mol
WT	–	−50.0	−719.8
Gly30Val + ΔArg31–Met33 (line D)	Gly28Val + ΔArg29–Met31	−51.0	−768.1
ΔIle32 (line G)	ΔIle30	−53.5	−814.4
ΔVal23–Thr24 (line J)	ΔVal21–Thr22	−50.3	−873.9
ΔVal27–Gly29 (line O)	ΔVal25–Gly27	−49.4	−748.7
Val23Met (line Q)	Val21Met	−53.0	−822.6
Val23Met + Gly30Ser (line R)	Val21Met + Gly28Ser	−49.4	−772.5
Thr24Pro (line T)	Thr22Pro	−51.6	−743.7

## Data Availability

The original contributions presented in this study are included in the article/Supplementary Material. Further inquiries can be directed to the corresponding author.

## Supplementary Materials

The following supporting information can be downloaded at: https://www.mdpi.com/article/10.3390/ijms27135971/s1.

## Abbreviations

The following abbreviations are used in this manuscript:

AIC	Akaike information criterion
ATCC	American Type Culture Collection
AZT	azithromycin
BIC	Bayesian information criterion
CFU	colony-forming units
CIP	ciprofloxacin
CLSI CRO	Clinical and Laboratory Standards Institute ceftriaxone
cryo-EM	cryo-electron microscopy
EF-G	elongation factor G
EUCAST	European Committee on Antimicrobial Susceptibility Testing
GBVI	Generalized Born/Volume Integral
GEN	gentamicin
HLR	high-level resistance
KAN	kanamycin
MIC	minimum inhibitory concentration
mRNA	messenger RNA
PBS	phosphate-buffered saline
PEN	penicillin
RING-MaP	RNA interaction groups analyzed by mutational profiling
RMSE	root-mean-square error
RpsE	ribosomal protein S5
rRNA	ribosomal RNA
SPC	spectinomycin
TCE	total contact energy
TET	tetracycline
tRNA	transfer RNA
WHO	World Health Organization
