# Supplementary material for: Evidence for a Constrained Mutational Pathway to High-Level Spectinomycin Resistance in Neisseria: RpsE Loop 2 Mutations and Associated Growth Costs
Source: Int J Mol Sci. 2026 Jul 3;27(13):5971. doi: 10.3390/ijms27135971 (PMC13361928; doi:10.3390/ijms27135971)
Supplement: Supplementary file 1 [file ijms-27-05971-s001.zip › Supplementary Figure S1.pdf]

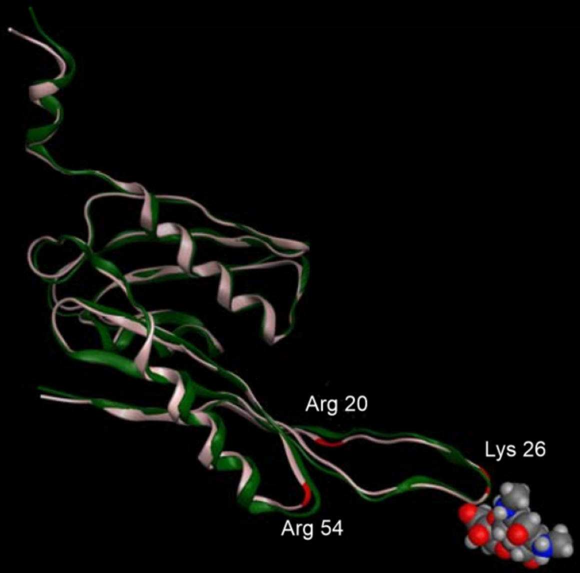

Supplementary Figure S1. Structural comparison of wild-type ribosomal protein RpsE (light beige) and the Val23Met variant (green; Val21Met in *E. coli* numbering) in complex with spectinomycin (space-filling model, red/blue). Residues Arg20 (Arg22 in *N. gonorrhoeae*), Lys26 (Lys28), and Arg54 (Arg56) are labelled to indicate positions showing the largest contact-energy differences between wild type and mutant.
